# Partial Inhibition of HO-1 Attenuates HMP-Induced Hepatic Regeneration against Liver Injury in Rats

**DOI:** 10.1155/2018/9108483

**Published:** 2018-04-15

**Authors:** Ning He, Jun-Jun Jia, Hai-Yang Xie, Jian-Hui Li, Yong He, Sheng-Yong Yin, Ruo-peng Liang, Li Jiang, Jing-feng Liu, Kang-di Xu, Zhi-hao Zhang, Lin Zhou, Shu-Sen Zheng

**Affiliations:** ^1^Department of Hepatobiliary and Pancreatic Surgery, First Affiliated Hospital, School of Medicine, Zhejiang University, Hangzhou 310003, China; ^2^Key Laboratory of Combined Multi-organ Transplantation, Ministry of Public Health, Hangzhou 310003, China; ^3^Collaborative Innovation Center for Diagnosis Treatment of Infectious Diseases, Hangzhou 310003, China; ^4^Department of Hepatobiliary and Pancreatic Surgery, The First Affiliated Hospital of Zhengzhou University, Zhengzhou, Henan Province 450052, China

## Abstract

We found better liver graft regeneration with hypothermic machine perfusion (HMP) compared with static cold storage (SCS) for the first time in our pilot study, but the underlying mechanisms are unknown. Upregulated heme oxygenase- (HO-) 1 expression has been reported to play a pivotal role in promoting hepatocyte proliferation. Here, we evaluated the novel role of HO-1 in liver graft protection by HMP. Rats with a heterozygous knockout of HO-1 (HO-1^+/−^) were generated and subjected to 3 h of SCS or HMP pre-half-size liver transplantation (HSLT) in vivo or 6 h of SCS or HMP in vitro; control rats were subjected to the same conditions (HO-1^+/+^). We found that HSLT induced significant elevation of the HO-1 protein level in the regenerated liver and that HO-1 haplodeficiency resulted in decreased proliferation post-HSLT. Compared with SCS, HMP induced significant elevation of the HO-1 protein level along with better liver recovery, both of which were reduced by HO-1 haplodeficiency. HO-1 haplodeficiency-induced decreased proliferation was responsible for the attenuated regenerative ability of HMP. Mechanistically, HO-1 haploinsufficiency resulted in suppression of hepatocyte growth factor (HGF)/Akt activity. Our results suggest that inhibition of HO-1 mitigates HMP-induced liver recovery effects related to proliferation, in part, by downregulating the HGF-Akt axis.

## 1. Introduction

The advent of successful liver transplantation, which was initially performed by Starzl et al., shifted the hepatic failure paradigm from an end-stage disease to a treatable condition with meaningful long-term survival, but it also created a new dilemma in that organ supply cannot meet liver transplantation demands [1, 2]. Better organ preservation methods, including the use of HMP, have expanded the donor pool to meet the growing organ demand with the use of marginal grafts.

HMP is an attractive option for avoiding a long warm ischaemia time and minimizing graft metabolic requirements while providing metabolic and oxygenation support. Although broadly implemented in renal transplantation, HMP remains investigational in clinical liver transplantation, with several animal studies showing that liver HMP is safe and, in most cases, superior to simple SCS, but the underlying mechanisms remain speculative [3]. Enhanced hepatocyte proliferation may result in better recovery after cold ischaemic injury and allow partial liver transplantation from donor corpses or living donors [4]. We found better liver graft regeneration with HMP compared with SCS for the first time in our pilot study. Therefore, we sought to determine the underlying mechanisms involved.

HO-1 is a ubiquitously expressed inducible enzyme that degrades haem to carbon monoxide (CO), biliverdin, and Fe^2+^. It participates in maintaining intracellular homeostasis and plays an important role in protecting cellular organization by reducing oxidative damage, inhibiting cell apoptosis, and attenuating the inflammatory response [5]. Increasing evidence suggests that HO-1 may play a role in protecting against various proliferative disorders, including transplant vascular stenosis, transplant rejection, and ischaemia-reperfusion (IR) injury among many others [6]. Relevant to the studies presented here, inhaled CO at low, nontoxic concentrations has been shown to accelerate liver regeneration in a mouse model of partial hepatectomy [7]. However, no studies have explored the role of HO-1 in recovering liver graft regeneration via HMP, which is the objective of our study.

Among the numerous growth factors with important roles in liver regeneration, HGF is the most notable, which is released from stellate cells [7]. Two additional key factors, IL-6 and TGF-*β*, have also been reported to be relevant for priming hepatocytes to proliferate [8] as their intracellular signals mediate hepatocyte proliferation through factors such as p-Akt and p-Erk mitogen-activated protein kinases [8].

Here, we performed HMP in control (HO-1^+/+^) rats and in rats with a heterozygous knockout of the HO-1 gene (HO-1^+/−^) in an in vivo HSLT model with 6 hours (h) of preservation in vitro and analysed the liver regeneration profile according to the activity of the HO-1-regulated signalling pathways. We found that a significant elevation of the HO-1 protein level and better liver recovery were induced by HMP both in vivo and in vitro, which were reduced by HO-1 haplodeficiency. HO-1 haplodeficiency induced decreased proliferation, but not apoptosis, and attenuated the regenerative ability of HMP both in vivo and in vitro. Mechanistically, HO-1 haploinsufficiency resulted in suppression of HGF/Akt activity. Our results suggest that liver graft protection by HMP at least partly involves HO-1-induced recovery of graft regeneration through the HGF/Akt signalling pathway in vivo and in vitro. To the best of our knowledge, this is the first time that HO-1 has been characterized as a novel positive regulator of the liver graft regeneration ability of HMP.

## 2. Material and Methods

### 2.1. Animals and Experimental Design

Adult male Sprague Dawley rats (250–300 g) were used in the experiments and were kept in environmentally controlled animal facilities. All experiments were approved by the Institutional Animal Care and Use Committee of Zhejiang University and were conducted in accordance with the ARRIVE (animal research: reporting in vivo experiments) guidelines (https://www.nc3rs.org.uk/arrive-guidelines).

Forty-eight HO-1^+/−^ rats (including 18 donors) and forty-eight control rats (HO-1^+/+^ rats obtained from the same breeding, including 18 donors) were randomly assigned to ten groups, including six in vivo groups (*n* = 12 in each group, including 6 donors) and four in vitro groups (*n* = 6 in each group), and each group was subjected to the following procedures:

In vivo: the orthotopic liver transplantation (OLT) group was treated with standard in situ HSLT following 45 min (the average operative time in our laboratory) histidine-tryptophan-ketoglutarate solution (HTK) perfusate (0–4°C) storage; the SCS groups were subjected to HSLT following 3 h of HTK perfusate (0–4°C) storage; the HMP groups were subjected to HSLT after the portal vein of the graft was connected to a home-made perfusion machine (portal vein velocity, 1.4 mL/min delivery; 4°C) [9], and the graft was perfused for 3 h with HTK solution.

In vitro: in the SCS group, the liver graft was placed into HTK perfusate (0–4°C) for 3 h or 6 h, and in the HMP group, the portal vein of the liver graft was connected to the perfusion machine and the graft was perfused for 3 h or 6 h with HTK solution.

### 2.2. Generation and Validation of TALEN-Mediated HO-1 Knockout Rats

**Figure 1:**
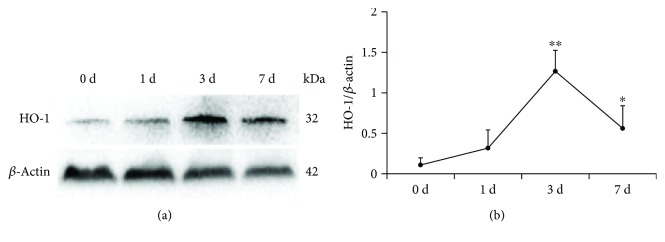
HO-1 expression pattern during liver regeneration progression. (a) Protein expression and (b) quantitative data (*n* = 3) of HO-1 during liver regeneration were analysed at the indicated time points via western blotting after HSLT. The data represent the mean ± SD. ^∗^ *p* < 0.05, ^∗∗^ *p* < 0.01 compared to the baseline (0 d group).

TALEN-mediated Hmox1 knockout rats were produced by Beijing View Solid Biotechnology, China. TALEN constructs in the TALEN-L and TALEN-R expression vectors were prepared with a HiSpeed plasmid midi kit (Qiagene), and the vectors were linearized with NotI. TALEN-L and TALEN-R mRNAs were transcribed in vitro using the MESSAGE mMACHINE® SP6 Kit (Invitrogen) with a T7 promoter. Zygotes of SD rats (*n* = 120) were injected with TALEN-L and TALEN-R mRNAs in M2 media (Millipore) using a FemtoJet micromanipulator (Eppendorf, Germany). After microinjection, the zygotes were transferred to pseudopregnant females. Tail-derived DNA from 2-week-old newborn rats was genotyped by sequencing PCR products amplified by the following primers: HO1-T4-sens (TTGACAGCTGGGCTGAAATGCAC) and Hmox1-T4-anti (TTCTGCGCAATCTTCTTCAGGAC). A 507 bp DNA fragment containing the TALEN target site was amplified, and the mutant Hmox1 alleles were confirmed by PCR sequencing to identify frameshift mutations. The mutant rats were mated with wild-type SD rats to obtain heterozygous Hmox1+/− rats (Figures 1(a) and 1(b)).

### 2.3. HSLT Model

#### 2.3.1. Donor Operation

The donor rat was fixed in the supine position and anaesthetized with 4% chloral hydrate through intraperitoneal administration. The left triangular ligament and left coronal ligament were isolated. Rat donor hemihepatic resection was completed by suture removal from the left lateral lobe and left middle lobe. An incision was made in the anterior wall of the common bile duct, and a stent was inserted. The right renal vein and the right adrenal vein were ligated following isolation of the inferior hepatic vein (IHVC), and the abdominal aorta was liberated below the kidney and cannulated with a catheter. Then, 100 *μ*l of heparin diluted in 2 ml of physiological saline was injected through the iliac vein. The left diaphragm was cut, the thoracic aorta was clamped, and the intrathoracic vena cava was transected to allow the flushing solution to rinse out. The liver was then perfused with approximately 10 ml of physiological saline solution at 0–4°C (2.5 ml/min) through the abdominal aorta until the liver became pale. The IHVC was divided, the distal pyloric vein was ligated, and the portal vein and suprahepatic inferior vena cava (SHVC) were severed. Then, the liver was removed and preserved in ice-cold normal saline at 0–4°C.

#### 2.3.2. Recipient Liver Resection

After fasting for 12 h before the operation, the rats were anaesthetized with 4% chloral hydrate via intraperitoneal administration. The left inferior phrenic vein and the proper hepatic artery were ligated and severed, and the common bile duct was transected proximal to the liver hilum. A thin rubber band was inserted behind the SHVC for traction. The recipient rat began to experience the anhepatic phase after the portal vein and IHVC were ligated. Then, 2 ml of normal saline was injected through the trifurcation where the portal vein was divided into the right and left branches. The IHVC was clamped with Satinsky forceps while the inserted rubber band was gently pulled, and then the liver of the recipient rat was removed. Then, the end of the IHVC was everted over a homemade cuff body with a similar size to that of a KAMADA and fixed to it. The same method was used for the portal vein.

#### 2.3.3. Liver Implantation

The donor liver was removed from the iced saline bath and placed in the orthotopic position. The donor rat SHVC was end-to-end anastomosed to the recipient rat SHVC with a running suture. A circumferential suture was used to complete the anastomosis after the cuffed portal vein was inserted into the recipient rat to end the anhepatic phase. Then, the same cuff procedure was conducted for the IHVC and the bile duct. The abdominal incision was closed with a continuous suture to complete the operation.

### 2.4. Sample Collection

At 0, 1, 3, and 6 h during the perfusion process in the in vitro groups and 1, 3, and 7 days (d) after HSLT in the in vivo groups, 2 mL of perfusate and plasma samples were collected from the in vitro and in vivo groups to analyse liver function. After 6 h of preservation in vitro and 7 d after HSLT in vivo, liver tissues were obtained and fixed in 10% neutral formalin for histological and immunohistochemical analyses. The livers were collected to determine the regeneration ratios (RRs, equal to the ratio of graft weight before HSLT to graft harvest weight at 7 d after HSLT) of the in vivo groups, and the liver tissues of all in vitro and in vivo groups were stored at −80°C for further experimental analysis.

### 2.5. Histopathologic Examination and Liver Function Tests

The liver tissues were excised, fixed in 4% paraformaldehyde, and embedded in paraffin. Then, 3 *μ*m liver sections were stained with haematoxylin and eosin after deparaffinization and hydration for morphological examination and routine immunofluorescent staining of Ki67 (Abcam, Cambridge, MA). Fatty acid binding protein (Fabp)-1 was analysed using the Fabp-1 ELISA Assay Kit (R&D systems, Minnesota, USA).

### 2.6. TUNEL Assay

Identification of hepatocyte apoptosis was conducted by terminal dUTP nick-end labelling (TUNEL) assay using an In Situ Cell Death Detection Kit, POD (Roche, Basel, Switzerland). Apoptotic hepatocytes were examined by fluorescence microscopy at a magnification of 200x.

### 2.7. Real-Time Quantitative PCR

Total RNA was extracted from liver graft tissues via TRIzol reagent (Thermo Fisher Scientific, Waltham, USA). Quantitative PCR was performed using a TaqMan instrument with an SYBR Green PCR kit (Takara Bio, Japan). The gene expression levels of caspase 3, bcl-2, bax, and P27 were quantified using a 7500 Fast Real-Time PCR instrument according to the manufacturer's instructions. The reactions took place in a 384-well plate at 95°C for 30 s, followed by 40 cycles of 95°C for 5 s, and 60°C for 30 s. Comparative CTs were calculated for relative quantification.

### 2.8. Quantitation of HGF, IL-6, and TGF*β*

For the quantitation of HGF, IL-6, and TGF*β*, liver lysates were adjusted to equal protein concentrations and used in the rat HGF IL-6 and TGF*β* ELISA Assay Kit (R&D systems, Minnesota, USA) following standard protocols.

### 2.9. Western Blotting

Protein was extracted from liver tissues using RIPA Lysis Buffer (Beyotime Institute of Biotechnology, China). After separation by SDS-polyacrylamide gel electrophoresis, soluble protein was transferred to nitrocellulose membranes. After blocking, the membranes were incubated overnight with primary antibodies against HO-1 (1 : 500; Cell Signaling, Danvers, MA), p-Akt (1 : 1000; Cell Signaling, Danvers, MA), t-Akt (1 : 1000; Cell Signaling, Danvers, MA), p-Erk1/2 (1 : 1000; Cell Signaling, Danvers, MA), t-Erk1/2 (1 : 1000; Cell Signaling, Danvers, MA), GAPDH (1 : 1000; Abcam, Cambridge, MA), and *β*-actin (1 : 1000; Abcam, Cambridge, MA). After thorough washes, the membranes were incubated with horseradish peroxidase-conjugated secondary antibodies and then subjected to an ECL kit (Pierce Biotechnology, Rockford, USA).

### 2.10. Statistical Analysis

All data were expressed as the mean ± SD. The statistical analysis was performed using an unpaired, two-tailed Student *t*-test (two groups) or one-way analysis of variance (ANOVA) followed by Dunnett's T3 for multiple comparisons. A *p* value less than 0.05 was considered statistically significant.

## 3. Results

### 3.1. HSLT Induces Significant Elevation of the HO-1 Protein Level in the Regenerated Liver

The reduced-size liver transplantation experimental model is commonly used to study liver regeneration. Here, we used an HSLT rat model to investigate the regenerative role of HO-1 in HMP. To explore the function of HO-1 in liver regeneration, we first examined whether HSLT could induce HO-1 in the regenerated liver. At the early stage post-HSLT, no obvious change was observed in the protein levels of HO-1 (a small increase on day 1). HO-1 expression increased by approximately 11.6-fold and 5.2-fold from 0 d to 3 and 7 d post-HSLT, suggesting possible involvement of HO-1 in the process of liver regeneration in HSLT (Figure 1).

### 3.2. HO-1 Haplodeficiency Results in Decreased Proliferation Post-HSLT

**Figure 2:**
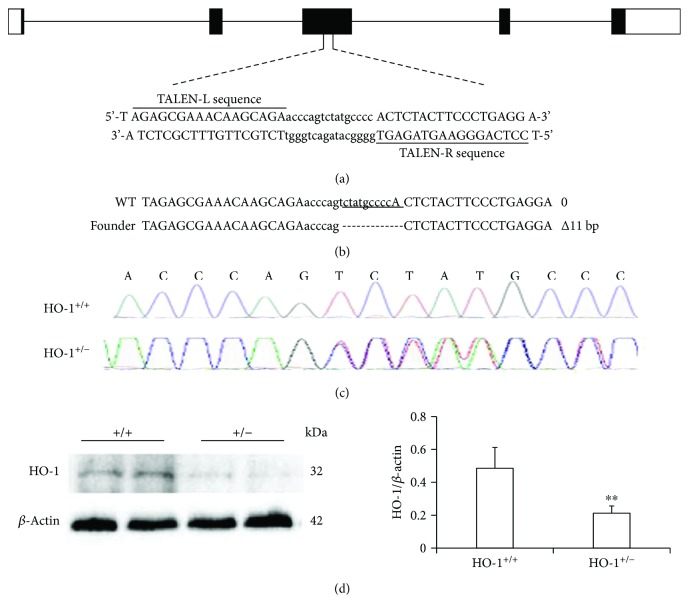
Generation of HO-1-haploinsufficient (HO-1^+/−^) rats. (a) Schematic of the Hmox1 genetic locus and target site of TALENs. (b) Representative results of DNA sequencing from WT (+/+) and heterozygous rats (+/−). (c) Sequencing chromatograms showing the 11 bp deletion in the heterozygous (HO-1^+/−^) sample. (d) Representative western blots (left) and statistical analysis (right) showing HO-1 expression in WT and HO-1^+/−^ livers under basal conditions (*n* = 8). The data represent the mean ± SD. ^∗∗^ *p* < 0.01.

**Figure 3:**
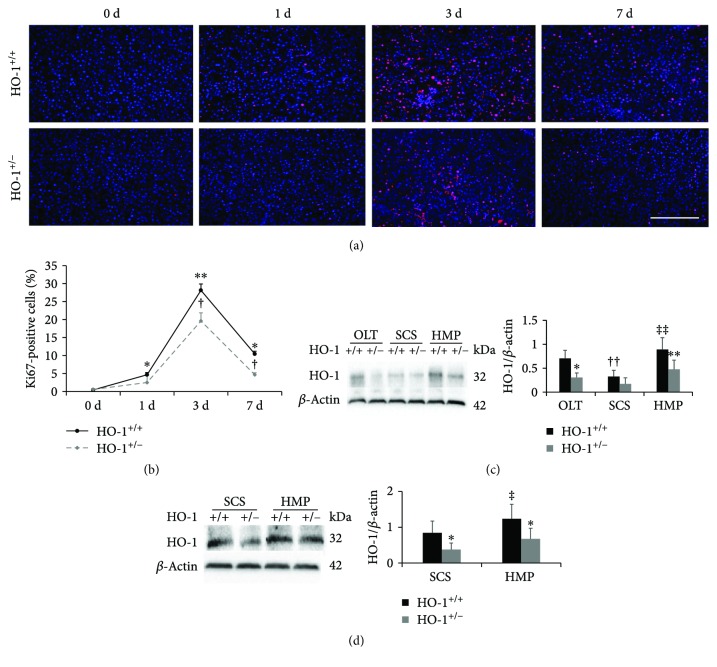
HO-1 haplodeficiency results in decreased proliferation and suppresses HMP-induced elevation of HO-1. (a) Representative sections from HO-1^+/−^ and WT (HO-1^+/+^) rats at the indicated time points after HSLT (*n* = 3; scale bar: 200 *μ*m). The liver sections were stained with anti-Ki67 (red) and were counterstained with DAPI (blue). (b) Quantification of the percentage of Ki67-labelled nuclei. (c) Protein expression and quantitative data (*n* = 6) of HO-1 in liver tissues were analysed on day 7 in vivo and (d) at 6 h in vitro via western blotting. The data represent the mean ± SD. (b) ^∗^ *p* < 0.05, ^∗∗^ *p* < 0.01 versus HO-1^+/+^ group at 0 d; ^†^ *p* < 0.05 versus HO-1^+/+^ rats of the same experimental group; (c, d) ^∗^ *p* < 0.05, ^∗∗^ *p* < 0.01 versus HO-1^+/+^ rats of the same experimental group; ^††^ *p* < 0.01 compared with OLT HO-1^+/+^ group; ^‡^ *p* < 0.05, ^‡‡^ *p* < 0.01 compared with SCS HO-1^+/+^ group.

Next, we aimed to determine the influence of knocking down HO-1 expression on liver regeneration and HO-1 expression changes induced by HMP. First, we induced global knockout of the HO-1 gene in rats (Figures 2(a)–2(d)). No HO-1^−/−^ rats were born, probably due to a developmental defect. Under basal conditions, HO-1^+/−^ rats grew normally and appeared healthy, with a similar liver structure and morphology compared to the livers of control rats (Supplementary Fig.  1); however, a 43% decrease in HO-1 protein expression was found in HO-1^+/−^ rat livers compared to that in control rat livers (Figure 2(d)). To further investigate the function of HO-1 in LR, we performed HSLT on HO-1^+/−^ rats and assessed cell proliferation in the regenerating liver by Ki67 labelling. Consistent with HO-1 protein expression post-HSLT in control rats, hepatocyte proliferation increased by approximately 63-fold and 23-fold from 0 d to 3 and 7 d post-HSLT, respectively, and HO-1^+/−^ significantly suppressed this increased proliferation at the same time points of 3 and 7 d, indicating a partial contribution of HO-1 to liver regeneration post-HSLT (Figures 3(a) and 3(b)).

### 3.3. HO-1 Haplodeficiency Suppresses HMP-Induced Elevation of HO-1 Protein Levels Both In Vivo and In Vitro

**Figure 4:**
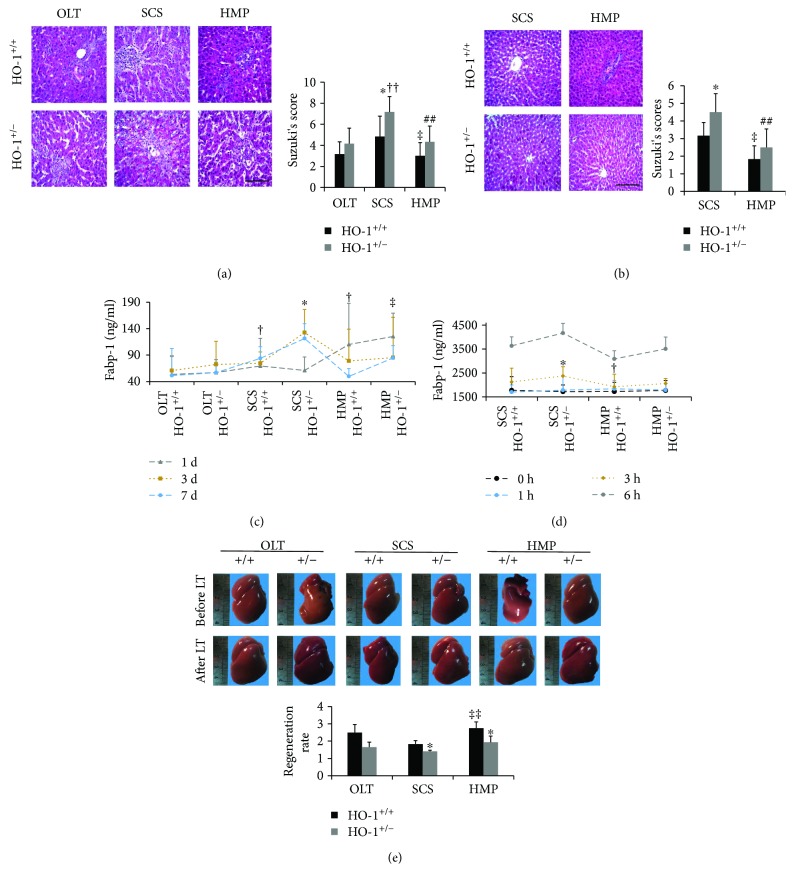
HO-1 haplodeficiency attenuates the liver recovery effects of HMP. (a) Histological analysis of livers from HO-1^+/−^ and WT (HO-1^+/+^) rats on day 7 in vivo and (b) at 6 h in vitro by haematoxylin and eosin staining (*n* = 6; scale bar: 200 *μ*m). (c) L-FABP was analysed by ELISA at the indicated times in vivo and (d) in vitro (*n* = 6). (e) Representative livers from rats before and 7 days after HSLT, indicating the differences in liver size and regeneration ratios (resected/regenerated weight), which were calculated to estimate the extent of liver regeneration between HO-1^+/−^ and WT (HO-1^+/+^) rats (*n* = 6). The data represent the mean ± SD. (a, b, e) ^∗^ *p* < 0.05 versus HO-1^+/+^ rats of the same experimental group; ^††^ *p* < 0.01 compared with OLT HO-1^+/−^ group; ^‡^ *p* < 0.05, ^‡‡^ *p* < 0.01 versus SCS HO-1^+/+^ group; ^##^ *p* < 0.01 versus SCS HO-1^+/−^ group. (c) ^∗^ *p* < 0.05 versus HO-1^+/+^ rats of the same experimental group at 3 d; ^†^ *p* < 0.05 versus HO-1^+/−^ rats of the same experimental group at 7 d; ^‡^ *p* < 0.05 versus SCS HO-1^+/−^ group at 7 d. (d) ^∗^ *p* < 0.05 versus HO-1^+/+^ rats of the same experimental group at 6 h; ^†^ *p* < 0.05 versus SCS HO-1^+/+^ group at 6 h.

We next tested the in vivo and in vitro expression of HO-1 induced by HMP. To explore the long-term effects of HMP, we selected the time point of 7 d in vivo. The rats in the in vivo experiment endured 3 h of HMP or SCS prior to HSLT, and we evaluated histopathological, functional, and proliferative indicators in vitro following 3 h of HMP or SCS but did not find significant results (Figure 4(d), Supplementary Fig.  2). Therefore, we extended the time in vitro to 6 h. We found a significant reduction in the HO-1 level in the SCS group compared with that in the OLT group, which was reversed by HMP treatment in vivo (Figure 3(c)). The same trend was observed in vitro between the SCS and HMP groups (Figure 3(d)). In addition, compared with the control rats, the HO-1^+/−^ rats showed markedly attenuated HO-1 expression. These results indicated that HO-1 may play an important role in HMP.

### 3.4. HO-1 Haplodeficiency Attenuates the Liver Recovery Effects of HMP Both In Vivo and In Vitro

Based on our observation of increased HO-1 expression during HMP, we next tested whether HMP-induced HO-1 participated in protection against liver damage induced by ischaemia and IR injury. Evidence suggests that liver function recovery is delayed during cold storage [10]. As expected, histological changes were observed in the SCS group versus the OLT group with HO-1^+/−^ rats reaching statistical significance. A larger area of vacuolation was observed in SCS liver samples together with sinusoidal congestion. Blinded pathologists scored these groups based on Suzuki's scores and reported lower scores in the HMP group than those in the SCS group in vivo and in vitro (Figures 4(a) and 4(b)), and HO-1 knockdown was related to increased scores. The levels of L-FABP, a sensitive marker of hepatocyte injury [11, 12], were decreased in the HMP group compared to those in the SCS group 7 d after HSLT in vivo and after 6 h of preservation in vitro, and HO-1 haplodeficiency resulted in increased L-FABP levels (Figure 4(d)). Additionally, regeneration ratios (RR) were calculated to estimate the extent of liver regeneration. We observed a higher RR (resected/regenerated weight) 7 d after HSLT in the HMP group compared to that in the SCS group (Figure 4(e)), and the HO-1^+/−^ mice had significantly greater RRs. Overall, these results suggested that HO-1 haplodeficiency attenuates the liver recovery effects of HMP related to hepatic injury as well as liver mass recovery effects both in vivo and in vitro.

### 3.5. HO-1 Haplodeficiency Partly Prevents HMP-Induced Proliferation but Not Apoptosis

**Figure 5:**
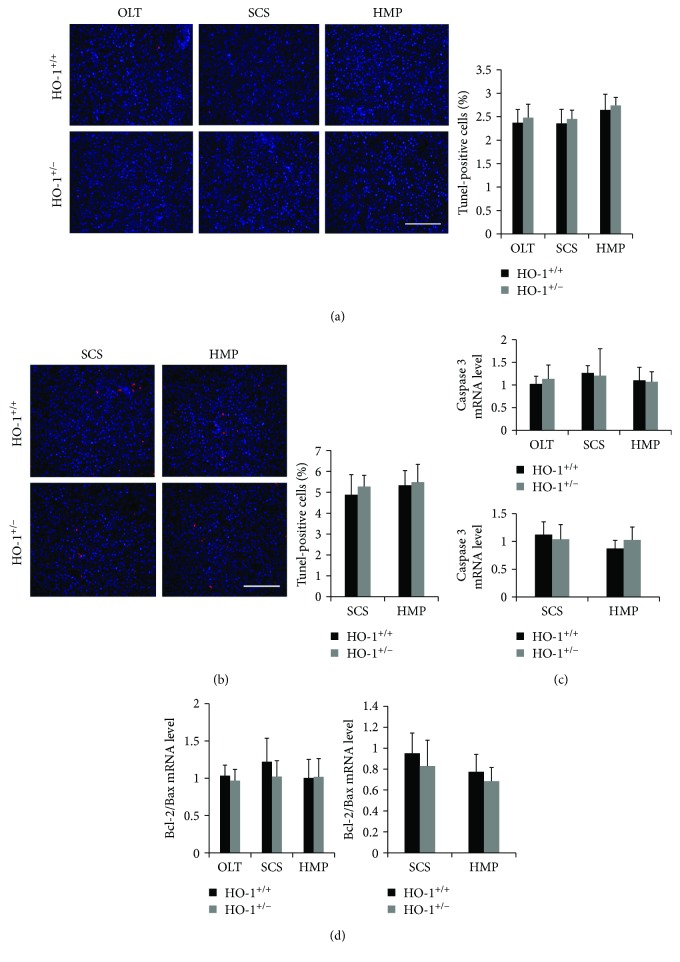
Apoptosis failed to explain the protective role of HO-1 in HMP. (a) Identification of hepatocyte apoptosis was conducted on liver sections on day 7 in vivo and (b) at 6 h in vitro by the terminal dUTP nick-end labelling (TUNEL) assay (*n* = 6; scale bar: 200 *μ*m), and the percentage of TUNEL-labelled nuclei was quantified. (c) Transcriptomic (qRT-PCR) analysis of caspase 3 and (d) Bcl-2/Bax in liver tissues from HO-1^+/−^ and WT (HO-1^+/+^) rats on day 7 in vivo and at 6 h in vitro (*n* = 6). The data represent the mean ± SD.

Previous studies have demonstrated that HO-1 protects against acute liver injury by inhibiting hepatocyte apoptosis [13]. Therefore, we first examined whether HO-1 ameliorates hepatocyte apoptosis in HMP-induced liver graft protection. No significant differences were found in the percentage of the TUNEL(+) hepatocytes among all groups in vivo and in vitro (Figures 5(a) and 5(b)). Similarly, the same trend was observed in the mRNA levels of caspase3 and the bcl2/bax ratio, which are indicators of cell apoptosis related to HO-1 [14] (Figures 5(c) and 5(d)).

**Figure 6:**
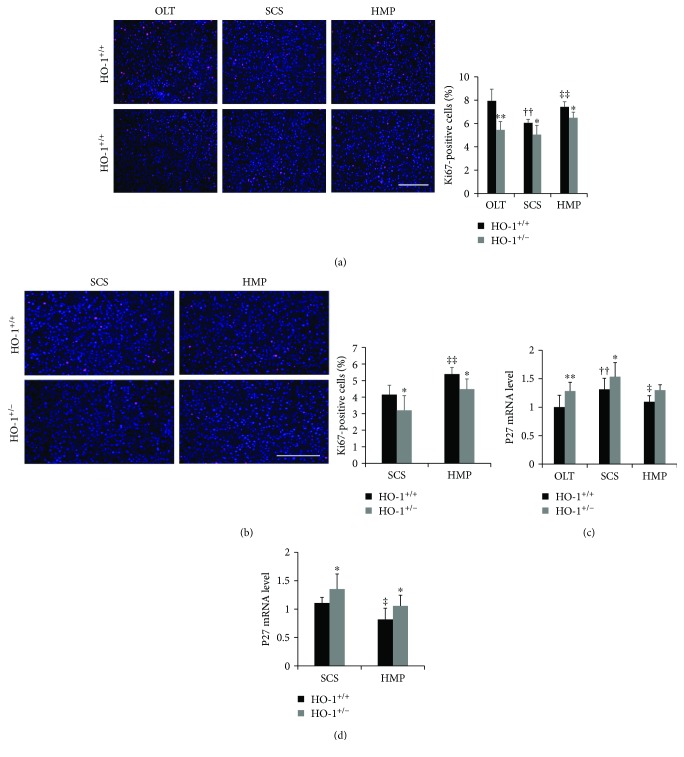
HO-1 haplodeficiency partly prevents HMP-induced proliferation. (a) Representative sections and quantification of the percentage of Ki67-labelled nuclei from HO-1^+/−^ and WT (HO-1^+/+^) rats on day 7 in vivo and (b) at 6 h in vitro (*n* = 6; scale bar: 200 *μ*m). The liver sections were stained with anti-Ki67 (red) and were counterstained with DAPI (blue). (c) Transcriptomic (qRT-PCR) analysis of P27 in liver tissues from HO-1^+/−^ and WT (HO-1^+/+^) rats on day 7 in vivo and (d) at 6 h in vitro (*n* = 6). The data represent the mean ± SD. ^∗^ *p* < 0.05, ^∗∗^ *p* < 0.01 versus HO-1^+/+^ rats of the same experimental group; ^††^ *p* < 0.01 compared with OLT HO-1^+/+^ group; ^‡^ *p* < 0.05, ^‡‡^ *p* < 0.01 compared with SCS HO-1^+/+^ group.

As apoptosis failed to explain the protective role of HO-1 in HMP, we next focused on the hepatocyte proliferative response and cell cycle regulators modulated by HO-1. We evaluated hepatocyte proliferation by staining of Ki67. We found a significantly reduced number of Ki67(+) hepatocytes in the SCS group compared with that in the OLT group, which was reversed by HMP treatment in vivo (Figure 6(a)). The same trend was observed in vitro between the SCS and HMP groups, and HO-1 haplodeficiency attenuated the proliferation response (Figure 6(b)). Furthermore, we evaluated the expression of P27Kip1 (P27), which is a known cell cycle inhibitor. The mRNA level of P27 was significantly increased in the SCS rats compared with that in the OLT rats, but this increase was blunted in the HMP rats in vivo. Similarly, the expression of P27 was obviously decreased in the HMP group compared with that in the SCS group in vitro. Knockdown of HO-1 significantly increased the suppression of P27 (Figures 6(c) and 6(d)). These results suggested that HMP-induced proliferation, but not apoptosis, is responsible for its better protection against liver injury compared to SCS, and HO-1 haplodeficiency prevents HMP-induced proliferation, but not apoptosis.

### 3.6. Partial Loss of HO-1 Inhibits Activation of the HGF/Akt Signalling Pathway

**Figure 7:**
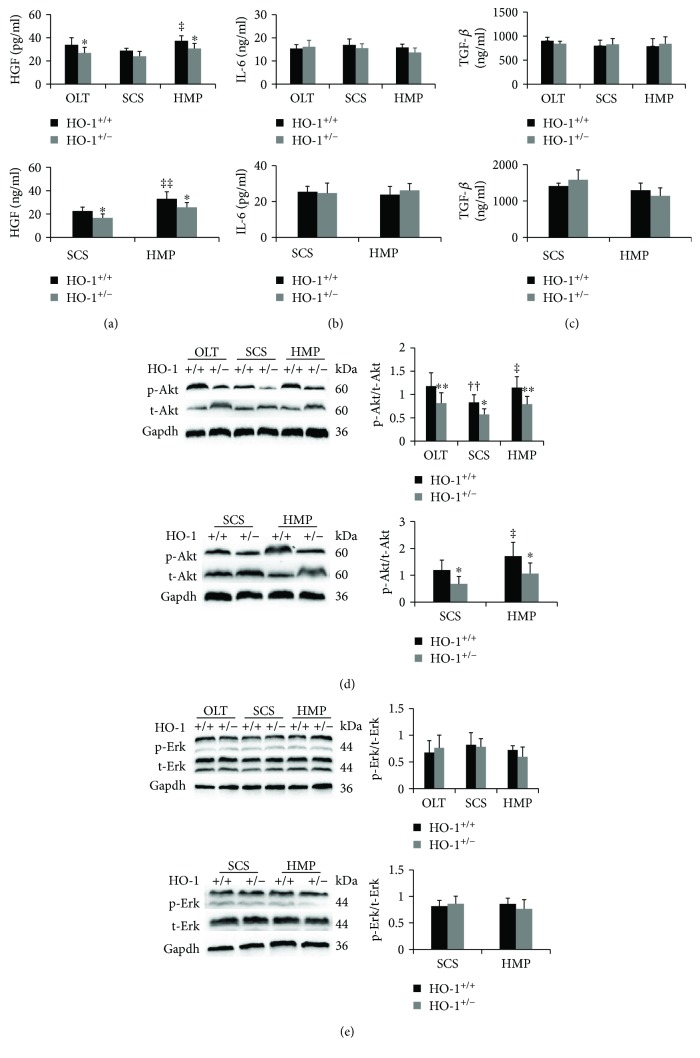
Partial loss of HO-1 inhibits activation of the HGF/Akt signalling pathway. (a) HGF, (b) IL-6, and (c) TGF-*β* were analysed by ELISA on day 7 in vivo and at 6 h in vitro (*n* = 6). (d) The protein expression and quantitative data of Akt and (e) Erk in liver tissues were analysed on day 7 in vivo and at 6 h in vitro via western blotting (*n* = 6). The data represent the mean ± SD. ^∗^ *p* < 0.05, ^∗∗^ *p* < 0.01 versus HO-1^+/+^ rats of the same experimental group; ^††^ *p* < 0.01 compared with OLT HO-1^+/+^ group; ^‡^ *p* < 0.05, ^‡‡^ *p* < 0.01 compared with SCS HO-1^+/+^ group.

Among the numerous growth factors with important roles in liver regeneration, HGF is the most notable, which is released from stellate cells [7]. The changes in HGF levels were consistent with HO-1 changes, with marked increases in the HMP group compared to the corresponding levels in the SCS group, which were significantly suppressed by HO-1 haplodeficiency (Figure 7(a)). We also measured IL-6 and TGF-*β* in liver tissue lysates, which have been reported to be relevant for priming hepatocytes to proliferate [8] (Figures 7(b) and 7(c)). However, no significant changes in the two mRNA levels were observed between the above-mentioned groups. Next, we determined the levels of the intracellular signals that mediate hepatocyte proliferation, including p-Akt and p-Erk mitogen-activated protein kinases [8]. The same trend as that for HGF was observed for p-Akt expression, while no significant results were found for p-Erk (Figures 7(d) and 7(e)). These findings suggest that inhibition of HO-1 mitigates liver regeneration in part by downregulating the HGF-Akt axis rather than IL-6 and TGF-*β* signalling.

**Figure 8:**
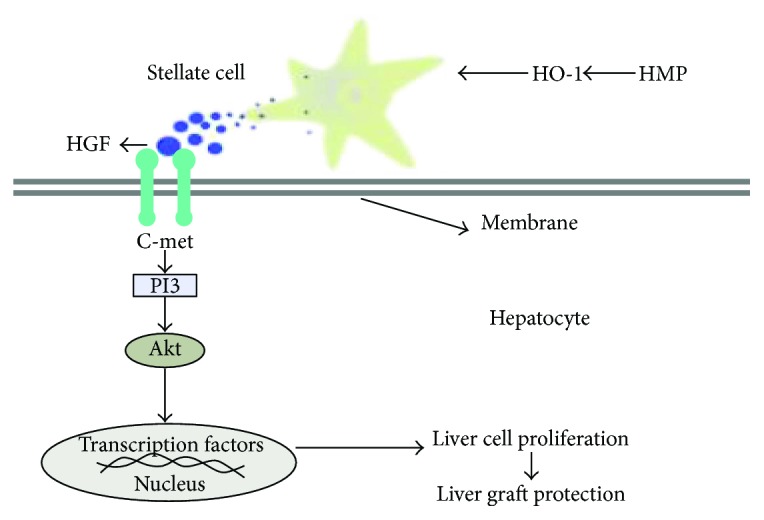
The probable mechanisms of the liver graft protective effects of HMP against liver injury. HMP: hypothermic machine perfusion; HO-1: heme oxygenase; HGF: hepatocyte growth factor.

Taken together, these results suggested that HO-1 probably exerts significant positive effects through HGF-Akt axis activation, but not through IL-6 and TGF-*β* signalling pathways, during HMP-induced liver graft recovery (Figure 8).

## 4. Discussion

In this study, we identified for the first time that HO-1 expression was upregulated with HMP versus SCS treatment of liver grafts; HO-1 participated in HMP's protection against hepatic injury and accelerated liver proliferation both in vivo and in vitro. HO-1-haplodeficient rats demonstrated significantly decreased HMP-induced proliferation, but not apoptosis, at least partly through inactivation of HGF/Akt signalling. These results suggest that induction of the HGF-Akt pathway by HO-1 is an adaptive mechanism during HMP-induced proliferation and that induction of HO-1 expression may be a new therapeutic target for liver injury during LT, especially in split liver transplantation (SLT).

In contrast to SCS, HMP with continuous delivery of metabolic substrates and flushing of metabolic by-products of the liver may provide better preservation of liver grafts and protection against ischaemia or IR injury [15, 16]. However, the underlying mechanisms remain unclear. A murine study suggested the protective role of hepatocyte proliferation against cold ischaemic injury in partial liver transplantation [4]. Consistent with this, we found better liver graft regeneration with HMP compared with SCS for the first time in our pilot study. Based on our observation of HO-1-induced promotion of hepatocyte proliferation [13], our first objective was to determine whether HO-1 exerts beneficial effects on liver regeneration after HSLT. Our results demonstrated that hepatocyte proliferation largely increased by 3 d and 7 d post-HSLT, which was consistent with HO-1 protein expression post-HSLT in the control rats. HO-1^+/−^ significantly suppressed this increased proliferation at the same time points of 3 and 7 d, indicating a partial contribution of HO-1 to liver regeneration post-HSLT. Therefore, we speculate that HO-1 expression levels may be relevant to HMP. Multiple studies have reported the tissue protective effects of HO-1 against organ IR injury [17]. Induction of HO-1 by CoPP in rat liver models of cold IR injury ex vivo and in orthotopic liver transplantation in vivo markedly preserved hepatic architecture and improved liver function [18]. Consistent with these studies, we found significantly reduced HO-1 levels and delayed RRs in vivo and functional and histopathological liver changes both in vivo and in vitro in the SCS group in response to ischaemia or IR injury, which were reversed by HMP treatment and were attenuated in HO-1 haplodeficiency.

HO-1 exerts significant beneficial effects on the maintenance of primary liver function due to potent antiapoptotic and proproliferative properties [7, 19]. Previous studies have provided evidence that the antiapoptotic and proproliferative effects of HO-1 involve an increased Bcl-2/Bax ratio and decreased caspase 3 [14] and p27-cell cycle inhibitor expression [20], respectively. Therefore, we speculate that the HO-1 expression level may be related to these effects. Indeed, our results revealed no significant differences in the percentage of the TUNEL(+) cells, mRNA levels of caspase 3, or the bcl2/bax ratio in vivo and in vitro. However, the HO-1 expression level was related to the number of Ki67(+) hepatocytes and p27 expression both in vivo and in vitro. More importantly, we provided evidence that partial loss of HO-1 could block HMP-induced hepatocyte proliferation in vivo and in vitro, which could significantly prevent the protective effects of HMP against liver injury.

Two signalling pathways are activated during the regenerative process, including a growth factor-mediated pathway in which HGF and TGF-*β* are released from hepatic stellate cells and a cytokine-regulated pathway involving complement factors C3a and C5a [21]. We systematically examined the two pathways and found that HGF increased the HO-1 expression level, while IL-6 and TGF-*β* did not show any differences among the treatment groups. These results are consistent with a recent study reporting that administration of exogenous CO, a product of haem degradation of HO-1, enhances early hepatocyte proliferation and preserves function by activating the HGF/Akt signalling pathway following partial hepatectomy [7]. Therefore, we evaluated phosphorylation of Akt, which is an intracellular signal downstream of HGF. Since Akt and Erk1/2 reflect common signalling pathways for the regulation of HO-1 and HGF expression [7, 22, 23], the influence of HO-1 on Erk1/2 phosphorylation was also investigated. In this study, HO-1 activated the Akt pathway, but not the Erk1/2 pathway, indicating that Akt phosphorylation, but not ERK1/2 phosphorylation, is involved in HO-1-mediated proliferation in vivo and in vitro. Therefore, inactivation of HO-1-induced downregulation of the Hgf/Akt axis in vivo and in vitro is involved in attenuating the liver recovery effects of HMP.

Interestingly, we tested functional and proliferative indicators in vitro after 3 h of HMP or SCS, which was consistent with the preservation time in vivo, and found insignificant results. However, the results changed substantially and reached significance when the preservation time was extended to 6 h. We speculate that some cytokines are generated locally or systematically during reperfusion after HMP and upregulate the production of HO-1. To corroborate this hypothesis, more studies are required.

In summary, our results suggest that inhibition of HO-1 mitigates HMP-induced liver recovery effects related to proliferation, but not apoptosis, in part by downregulating the HGF-Akt axis rather than IL-6 and TGF-*β* signalling in vivo and in vitro. Therefore, specific pharmacological targeting of HO-1 induction via HMP may have potential implications for the optimal preservation and reconditioning of allografts and may be potentially applicable in clinical practice.

## Supplementary Materials

Supplementary 1 Figure S1: liver histopathological presentations of HO^+/+^ (A) and HO-1^+/−^ (B) rats.

Supplementary 2 Figure S2: histopathological, functional, and proliferative indicators in rats in vitro after 3 h of HMP or SCS.
