# Supplementary figures and images for: Partial Inhibition of HO-1 Attenuates HMP-Induced Hepatic Regeneration against Liver Injury in Rats
Source: Oxid Med Cell Longev. 2018 Apr 15;2018:9108483. doi: 10.1155/2018/9108483 (PMC5925174; doi:10.1155/2018/9108483)

## Slide 1
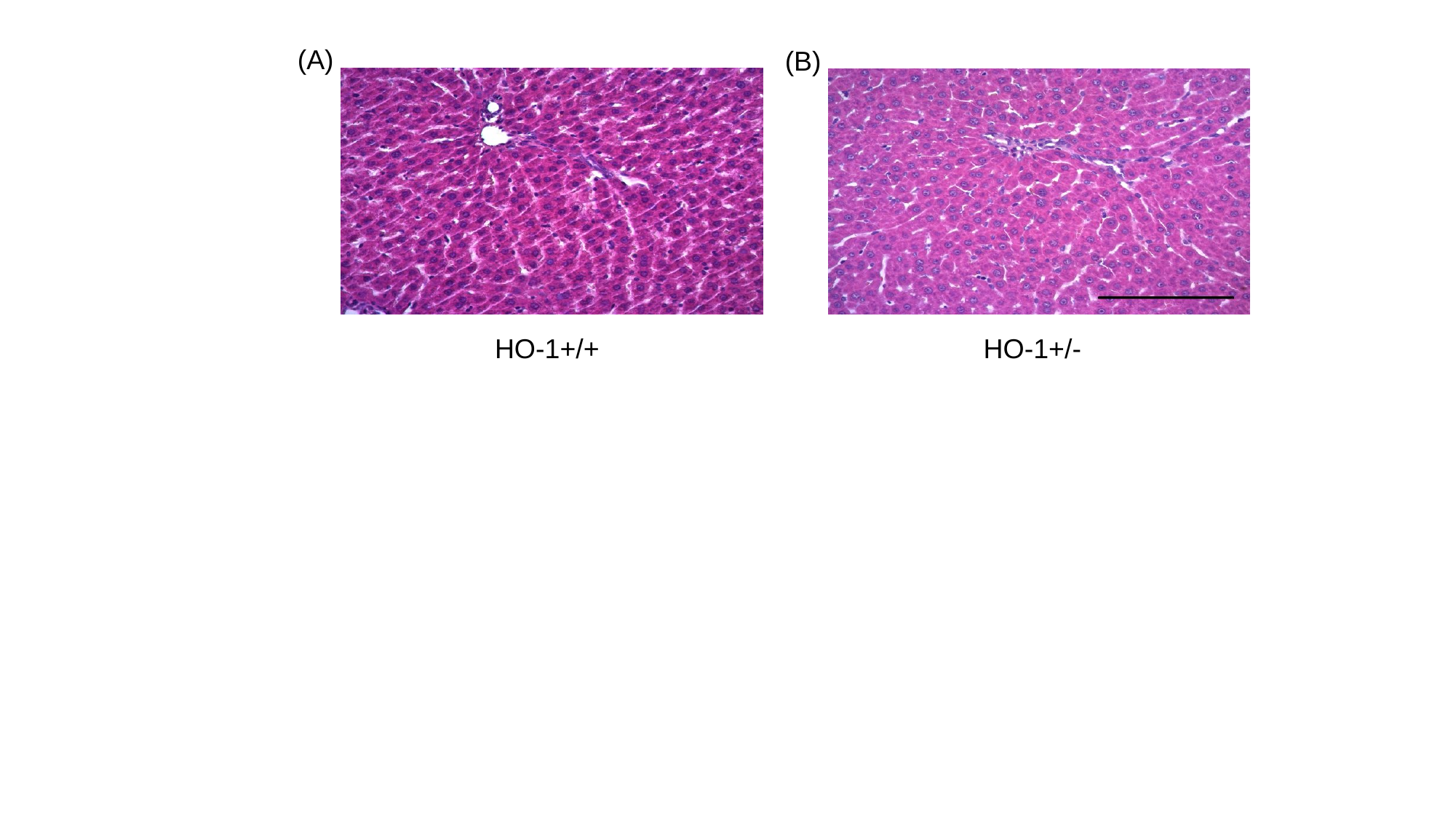

(A)
(B)
HO-1+/+
HO-1+/-

Supplement: Supplementary 1 — Figure S1: liver histopathological presentations of HO+/+ (A) and HO-1+/− (B) rats. [file 9108483.f1.pptx]

## Slide 1
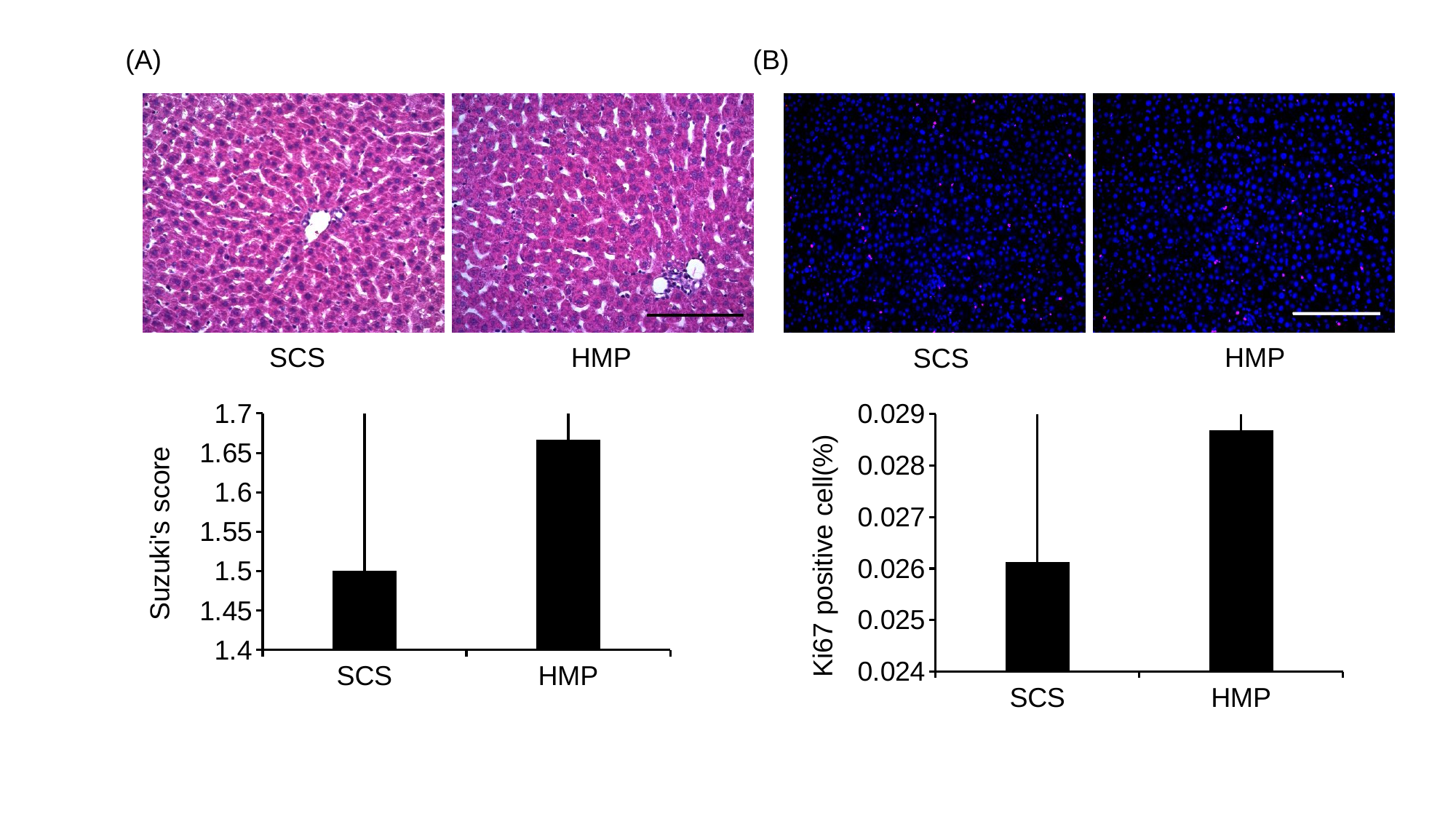

(A)
(B)
SCS
HMP
HMP
SCS
### Chart
| Category | |
|---|---|
| SCS | 0.02613226586453585 |
| HMP | 0.02868045955878346 |
### Chart
| Category | |
|---|---|
| SCS | 1.5 |
| HMP | 1.6666666666666667 |

Supplement: Supplementary 2 — Figure S2: histopathological, functional, and proliferative indicators in rats in vitro after 3 h of HMP or SCS. [file 9108483.f2.pptx]
